# Medical Women in the Army

**Published:** 1918-07-27

**Authors:** 


### MEDICAL WOMEN IN THE ARMY.

There are now a considerable number of medical women attached to the Royal Army Medical Corps for duty at Base hospitals at home and abroad, where they perform the same duties as commissioned medical officers. The circumstances of their enrolment were novel and unique; they responded nobly to the call of their country in its difficulties and accepted such conditions of service as were offered, in an unquestioning and confiding spirit. The experience of these ladies with the Colours, however, extending in some instances to over two years, has disclosed and accentuated marked differences in the conditions of service and treatment of medical women as compared with commissioned medical officers. Women serving at home receive neither the ration, the billeting allowance, nor the gratuity of £60 which is paid to temporary commissioned officers. They are also ineligible for the half-fare vouchers for railway journeys on leave, and they travel third class on duty. They also pay income tax as civilians. Women serving abroad receive the same pay as men and the same gratuity, but they receive neither the ration nor the billeting allowance.

These grievances are sufficiently serious, but they affect the individual rather than the national cause. The medical women have no rank! This is not only the greatest personal grievance, it is the greatest obstacle to the successful execution of any military duty. Rank is the only authority which is recognised in the Army, and the maintenance of a responsible position without it is an impossibility. The necessity of unadulterated rank for responsible persons in every department of the Army is now sufficiently established; after fifty years of evolution, hyphenated titles have at last been entirely eliminated from the Medical Services, and chaplains, post office, labour, and railway superintendents and managers are now captains, colonels, and generals. That rank is essential for the maintenance of responsible positions in the Army, whether filled by men or women, is beyond argument. We were therefore pained to learn that in the House on July 2, in reply to a question by Sir Robert Newman, the Financial Secretary to the War Office refused in the name of the Government to consider the granting of commissions to medical women serving in the Army.

There are, of course, difficulties in the execution of reforms. Public opinion, whether it be civilian or military, must first be educated; unconsidered changes are deprecated by the very conservative British Army, and the sudden apparition of a lady officer might be followed by unforeseen consequences. It is possible also that British women are not yet sufficiently emancipated to bear responsibility alone; doubtless many are, and it should be feasible to select these by a system of probationary service.

The opinion of the Army on the subject is not fully formed; there exists a certain ill-defined apprehension, but on the whole no prohibitive feeling. The branch of the Service most deeply concerned, the R.A.M.C., would not endure the command of a woman under present conditions, so that it would be necessary to limit the rank to that of temporary second-lieutenant, and adjust the pay by additional allowances; it would also be necessary to emphasise the temporary and emergent nature of the contract, which should end as soon as men became available. In the American Army, women doctors join the Medical Service with the rank of first-lieutenant, under conditions common to both sexes; Canadian nurses also wear badges of rank. Neither the men nor the women of the British Isles, however, have reached this stage of evolution, although at the recent rate of progress the goal should soon be attained.

